# Carbon Dots Synergizing with Phosphate Starch to Construct Dual Conduction Pathways for High-Performance Smart Packaging Films

**DOI:** 10.3390/ma18245644

**Published:** 2025-12-16

**Authors:** Xiaoxu Zhang, Chengguo Liu, Xiaoqin Yang, Qian Jiang, Can Liu, Ping Zhao, Jingyan Liu

**Affiliations:** 1 National Joint Engineering Research Center for Highly-Efficient Utilization Technology of Forestry Resources, Southwest Forestry University, Kunming 650224, China; 18183867795@163.com (X.Z.); liuchengguo@njfu.edu.cn (C.L.); yangxiaoqin@swfu.edu.cn (X.Y.); jqian202304@163.com (Q.J.); liucan@swfu.edu.cn (C.L.); 2 Jiangsu Co-Innovation Center of Efficient Processing and Utilization of Forest Resources, Jiangsu Key Lab for the Chemistry and Utilization of Agricultural and Forest Biomass, College of Chemical Engineering, Nanjing Forestry University, Nanjing 210037, China

**Keywords:** ellagic acid, carbon dots, phosphate starch, starch-based film, smart packaging

## Abstract

The advancement of smart packaging technology demands high-performance and sustainable sensing materials. While starch is a biodegradable natural polymer, its inherent high crystallinity restricts charge transport capability. This study developed a novel smart sensing film by incorporating ellagic acid-derived blue, fluorescent carbon dots (CDs) into phosphate starch (PS), which is rich in phosphorus. The effects of silver ions (Ag^+^), sodium carboxymethyl cellulose (CMC), and CDs on the film properties were systematically investigated. Results indicate that CDs act as flexible nano-crosslinkers, forming hydrogen bonds with PS molecular chains and effectively balancing strength and toughness—achieving a tensile strength of 5.1 MPa and an elongation at break of 24.1%. Phosphorus, in synergy with CDs, facilitates an efficient dual conduction pathway for ions and electrons: phosphate groups enable ion transport, while the conjugated carbon cores of the CDs provide electron transport channels. This synergistic effect significantly reduces the film’s electrical impedance from 6.93 × 10^6^ Ω to 1.12 × 10^6^ Ω (a reduction of 84%) and enhances thermal stability, increasing the char residue from 1.1% to 18.3%. The PS/CDs composite film exhibits a strong linear current response to pH in the range of 2–7 (*R*^2^ = 0.9450), and shows enhanced discrimination between fresh orange juice (pH = 3.38) and spoiled orange juice (pH = 2.68), with a current change of 0.62 × 10^−5^ A. Moreover, the film exhibits strong blue fluorescence at 427 nm, with an intensity that shows a pronounced pH-dependent response. This study elucidates the mechanism by which phosphorus and CDs synergistically enhance the sensing performance of starch-based films, offering a new strategy for developing high-performance starch-based materials for smart packaging.

## 1. Introduction

Driven by the growing demand for food safety and quality monitoring, smart packaging systems capable of real-time sensing and information relay have attracted profound interest [1,2]. Among various sensing platforms, biopolymer films integrated with functional nanomaterials are particularly promising, owing to their sustainability, biodegradability, and tunable physicochemical properties [3,4]. Starch, an abundant and renewable polysaccharide, represents an attractive green substrate for packaging. Nevertheless, its inherent highly crystalline nature and poor charge transport capacity severely hinder its application in sensing devices [5,6,7].

To address these limitations, physical [8,9] and chemical [10,11] modification of starch have been widely investigated. Among various modifying agents, silver ions (Ag^+^) have garnered significant interest owing to their exceptional antibacterial properties and capacity to form cross-linked networks via coordination bonds. Although incorporating Ag^+^ into the starch matrix has been demonstrated to enhance the mechanical strength and thermal stability of the resultant films, the strong coordination with starch molecular chains typically restricts chain mobility, resulting in embrittlement. Furthermore, potential biotoxicity concerns hinder their direct application in food-contact materials [12,13]. On the other hand, sodium carboxymethyl cellulose (CMC), an anionic cellulose derivative, is frequently employed as a reinforcing agent for starch. The electrostatic interactions and hydrogen bonding between CMC and starch molecular chains facilitate the formation of a dense interpenetrating network, which markedly improves the tensile strength of the films [14,15]. However, this rigid network often induces severe embrittlement [16,17]. In addition, the inherent insulating nature of CMC impedes efficient charge transport, thereby constraining its potential for sensing applications.

In the pursuit of more balanced modification strategies, phosphate starch (PS) demonstrates enhanced hydrophilicity and improved ion transport capacity owing to the incorporation of phosphate groups, thereby offering a viable approach to addressing conductivity challenges. Meanwhile, carbon dots (CDs), an emerging class of carbon-based nanomaterials, have shown considerable promise in composite applications, attributed to their tunable surface functionalities, distinctive photoluminescence properties, and efficient electron transfer capabilities [18,19,20]. When synthesized from natural polyphenols such as ellagic acid, CDs can further impart heightened fluorescence performance and favorable biocompatibility to host materials [21,22,23]. The integration of CDs with PS is anticipated to yield a synergistic system that combines the ion-conductive benefits of PS with the electron-transport advantages of CDs. Moreover, the abundant surface functional groups on CDs can facilitate ductility cross-linking, contributing to an optimal balance between mechanical strength and toughness.

Based on the above background, this work systematically investigates the effects of three modifiers—Ag^+^, carboxymethyl cellulose (CMC), and carbon dots (CDs)—on the structure and properties of phosphate starch (PS) films. Emphasis is placed on the role of CDs as flexible nano-crosslinkers that interact with the PS matrix via hydrogen bonding, leading to a balanced strength–toughness performance. Furthermore, this study elucidates the critical contribution of the conjugated architectures formed between phosphate groups and CDs in establishing synergistic ion–electron transport channels, which significantly reduce film impedance and impart sensitive dual-mode current/fluorescence responses to pH variation. These findings provide a new design strategy and theoretical support for developing high-performance multifunctional starch-based sensing materials toward smart packaging applications.

## 2. Materials and Methods

### 2.1. Experimental Materials

Ultrapure water was obtained from a laboratory water purification system. Phosphate starch (PS), with a degree of substitution (DS) of 0.05–0.15 and a pH range of 5.6–7, was supplied by Aladdin Biochemical Technology Co., Ltd. (Shanghai, China). Sodium carboxymethyl cellulose (CMC, analytical grade), silver nitrate (AgNO_3_, analytical grade), ellagic acid (analytical grade), terephthalaldehyde (analytical grade), and methanol (analytical grade) were all purchased from Aladdin Biochemical Technology Co., Ltd. (China). Sodium hydroxide (NaOH, analytical grade) and sodium chloride (NaCl, analytical grade) were procured from Sinopharm Chemical Reagent Co., Ltd. (Shanghai, China). Glycerol (analytical grade) was sourced from Guangdong Guanghua Sci-Tech Co., Ltd. (Guangzhou, China). All chemicals were used as received without further purification.

### 2.2. Preparation of Ellagic Acid CDs

Carbon dots (CDs) were synthesized via a hydrothermal approach. Specifically, 0.1 g of ellagic acid, 0.1 g of terephthalaldehyde, and 0.1 g of NaOH were dissolved in 15 mL of methanol in a Teflon-lined autoclave. The reaction was conducted at 210 °C for 6 h to allow carbonization. After cooling to ambient temperature, the contents were homogenized by ultrasonication. The resulting mixture was filtered and adjusted to neutral pH, followed by further filtration to obtain a clear solution. This solution was transferred to a round-bottom flask and concentrated using a rotary evaporator operating at 55 °C and 120 rpm. The final carbon dots, obtained as a dry solid adhering to the flask walls, were stored for subsequent use.

### 2.3. Preparation of PS Composite Films

Composite films using phosphate ester starch (PS) as the matrix were prepared via the solution casting method. The basic film-forming solution for all samples consisted of 4 g PS, 1.4 g glycerol (35% by starch mass), and 96 mL ultrapure water.

(1) PS/AgNO_3_ composite film: 1.0 mL of an aqueous AgNO_3_ solution (10 mmol/L) was added to 100 mL of the basic film-forming solution. The mixture was stirred at room temperature for 30 min, followed by stirring in a 90 °C water bath for 1 h.

(2) PS/CMC composite film: 1.0 g of sodium carboxymethyl cellulose (CMC) was added to 100 mL of the basic film-forming solution and processed under the same stirring procedure.

(3) PS/CD composite film: 10 mg of carbon dots (CDs) was added to 100 mL of the basic film-forming solution and processed under the same stirring procedure.

The film-forming solutions from each series were cast into polytetrafluoroethylene (PTFE) Petri dishes (15 cm in diameter) and dried at 45 °C for 6 h to form free-standing films. All resulting films were designated according to a unified naming system as “PS/component-dosage”, consistent with the labels of their corresponding precursor solutions. The reported loadings of the modifiers are based on the optimal ratios identified through preliminary screening experiments.

### 2.4. Methods

The chemical structure and functional groups of the composite film were characterized by Fourier Transform Infrared Spectroscopy (FTIR, Nicolet iS50, Thermo Fisher Scientific, Waltham, MA, USA) using the KBr pellet method. Spectra were collected from 4000 to 400 cm^−1^ with a resolution of 4 cm^−1^ over 32 scans. Crystal structure was examined by X-ray diffraction (XRD) with CuKα radiation (λ = 0.1541 nm) operated at 40 kV and 40 mA. Diffraction patterns were recorded in the 2θ range of 5–50° at a scanning rate of 8°/min with a step size of 0.02°. The interlayer spacing was calculated using the Bragg equation. Surface elemental composition and chemical states were analyzed by X-ray Photoelectron Spectroscopy (XPS, Thermo Fisher Scientific K-Alpha, Waltham, MA, USA) with monochromated AlKα radiation (1486.6 eV). Binding energies were calibrated relative to the C 1s peak at 284.8 eV. Scanning electron microscopy (SEM) at 15 kV was used to observe the morphology and cross-sectional structure of films fractured in liquid nitrogen. Mechanical properties, including tensile strength and elongation at break, were measured on a universal testing machine (Instron 5967, Norwood, MA, USA) following GB/T 1040-2006. Specimens were cut into 80 mm × 15 mm strips and tested at a crosshead speed of 25 mm/min, with five replicates per group. Thermal stability was assessed by Thermogravimetric Analysis (TGA, Q50, TA Instruments, New Castle, DE, USA) under a nitrogen atmosphere, heating from 35 °C to 600 °C at 20 K/min. Surface wettability was evaluated by measuring the static contact angle of 4 μL ultrapure water droplets after 3 s using a contact angle goniometer (OCA20, DataPhysics Instruments GmbH, Filderstadt, Germany). Fluorescence properties were investigated by recording excitation and emission spectra on a fluorescence spectrophotometer (F-7000, Hitachi High-Tech Corporation, Tokyo, Japan). Electrochemical Impedance Spectroscopy (EIS) was performed with an electrochemical workstation (CHI760E, CH Instruments, Shanghai, China) over a frequency range of 0.1 Hz to 1 MHz. The pH response was evaluated via chronoamperometry by applying 0.3 mL of buffer solutions at varying pH values (1, 4, 7, 9, 13) onto the device surface and recording the steady-state current. Practical applicability was further verified by testing the device with 0.3 mL of fresh (pH = 3.38) and spoiled (pH = 2.68) orange juice using the same chronoamperometric method.

## 3. Results

### 3.1. Structure and Fluorescence of Ellagic Acid-Based Carbon Dots

**Figure 1:**
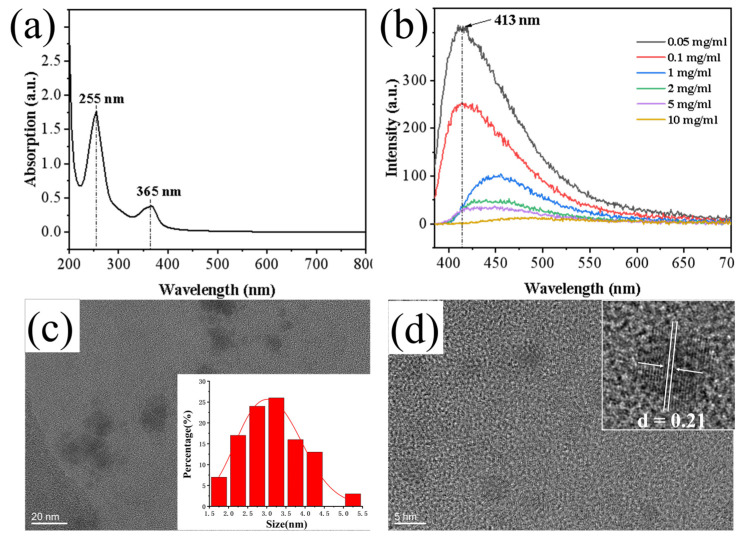
(**a**) UV absorption spectrum of CDs; (**b**) Fluorescence spectrum of CDs; (**c**) Size distribution histogram of CDs; (**d**) HRTEM image showing lattice fringes of CDs.

Carbon dots (CDs) were synthesized using ellagic acid as the carbon source. The UV-Vis absorption spectrum of the CDs exhibited a strong peak at approximately 225 nm, which is typically attributed to the π–π* transition of aromatic C=C bonds in sp^2^ hybridized carbon domains. A distinct characteristic peak at around 365 nm corresponds to the n–π* transition of C=O bonds (Figure 1a). These features indicate that the CDs possess a partially graphitized crystalline core alongside oxygen-containing functional groups (e.g., C=O, –OH), which generate surface states with a continuous energy distribution, enabling broad-band absorption from the ultraviolet to visible region. Fluorescence spectra showed that the fluorescence intensity of the CDs decreased with increasing concentration, suggesting fluorescence quenching due to aggregation at high concentrations (Figure 1b). High-resolution transmission electron microscopy (HRTEM) images and the corresponding size distribution analysis confirmed that the synthesized CDs were well-dispersed, quasi-spherical nanoparticles with a narrow size distribution and an average diameter of about 3.25 nm (Figure 1c). Clear lattice fringes with a spacing of 0.21 nm were observed (Figure 1d), indicating the presence of graphitic-like structures within the amorphous carbon framework.

### 3.2. Effect of Modifiers on the Mechanical Properties of PS Films

**Figure 2:**
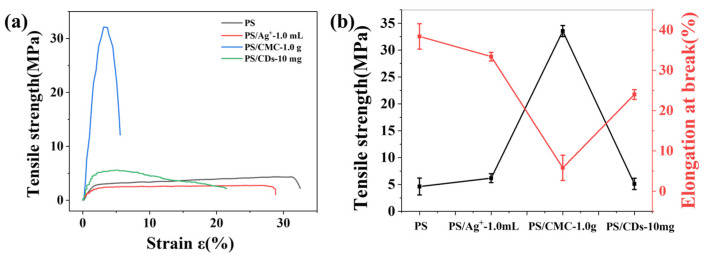
Tensile properties of starch-based films (**a**) Stress–strain curves; (**b**) Tensile strength and elongation at break.

The influence of silver ions (Ag^+^), sodium carboxymethyl cellulose (CMC), and carbon dots (CDs) on the mechanical properties of phosphate starch (PS) films was evaluated by stress–strain measurements (Figure 2). Pristine PS films showed characteristic ductile fracture behavior, with a tensile strength of 4.6 MPa and an elongation at break of 38.4%. The incorporation of phosphate groups disrupted intermolecular hydrogen bonding between starch chains via steric hindrance, resulting in enhanced film toughness.

Pure/unmodified starch films: typically exhibit relatively low strength and brittleness, with tensile strength generally ranging from approximately 5 to 25 MPa. Plasticized starch films (the most commonly studied systems) usually demonstrate tensile strengths between 2 and 15 MPa [24,25]. The incorporation of different modifiers resulted in marked variations in the mechanical properties of the composite films. The PS/Ag^+^ film exhibited an increased tensile strength of 6.2 MPa, while its elongation at break decreased to 33.4%. This behavior is attributed to coordination crosslinking between Ag^+^ and phosphate groups, which enhances stress transfer but restricts molecular chain mobility. The PS/CMC composite demonstrated the highest tensile strength (33.5 MPa), but its elongation at break dropped sharply to 5.8% (Figure 2a), resulting from electrostatic interactions and hydrogen bonding between CMC and PS chains that form a compact interpenetrating network. This structure improves stress transfer efficiency but severely limits chain slippage. In contrast, the PS/CDs composite achieved a balanced mechanical performance, with a tensile strength of 5.1 MPa and an elongation at break of 24.1% (Figure 2b). The abundant hydroxyl and carboxyl groups on the CDs surface form hydrogen bonds with PS molecular chains, serving as nanoscale flexible crosslinking points [25]. These points restrict excessive chain slippage while preserving adequate segmental mobility, thereby achieving an optimal strength–toughness balance. These characteristics render the PS/CDs composite film a promising candidate for smart packaging applications [26].

### 3.3. Effect of Modifiers on the Chemical Structure of PS Films

**Figure 3:**
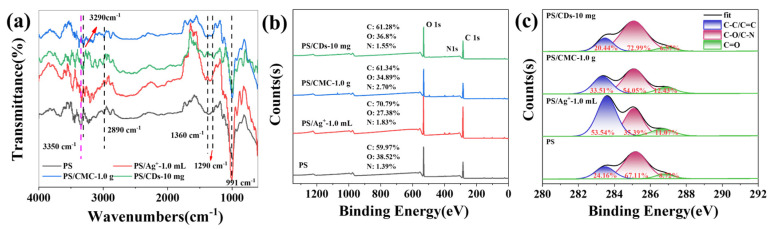
(**a**) FTIR of the PS composite film; (**b**) XPS survey scan of the PS composite film; (**c**) Content of carbon bonding states in the PS composite film (XPS).

The chemical structure of phosphate starch (PS) was analyzed by FTIR spectroscopy (Figure 3). The spectrum of PS exhibited characteristic starch absorption bands at ~3350 cm^−1^ (O–H stretch), ~2890 cm^−1^ (C–H stretch), and between 1150 and 1015 cm^−1^ (C–O/C–O–C vibrations), along with the emergence of a new peak at ~1290 cm^−1^, which is assigned to the P=O stretching vibration. Furthermore, spectral variations in the 1000–1100 cm^−1^ range suggest the formation of P–O–C vibrations. Both PS/CMC and PS/CDs composite films exhibited a red shift in the O–H stretching vibration peak to 3290 cm^−1^, indicating the formation of hydrogen bonds between PS and CMC or CDs, respectively [27,28]. This finding correlates well with the increased tensile strength and reduced elongation at break observed in mechanical tests.

XPS analysis provided further insight into the chemical composition changes in the composite films. The PS/CMC film showed an increase in C–C content from 24.16% to 33.51% and a rise in C=O content to 12.43% (Figure 3c), resulting from the incorporation of carboxyl and ether groups from CMC molecules. In contrast, the PS/CDs composite exhibited a significant increase in C–O content from 67.11% to 72.99%, accompanied by decreases in both C–C and C=O contents. This trend reflects the dominant role of oxygen-rich functional groups (e.g., hydroxyl and epoxy groups) on the carbon dot surfaces in shaping the interfacial chemical environment, leading to the formation of a dense hydrogen-bonding network with PS.

### 3.4. Effect of Modifiers on the Crystalline Structure and Thermal Stability of PS Films

**Figure 4:**
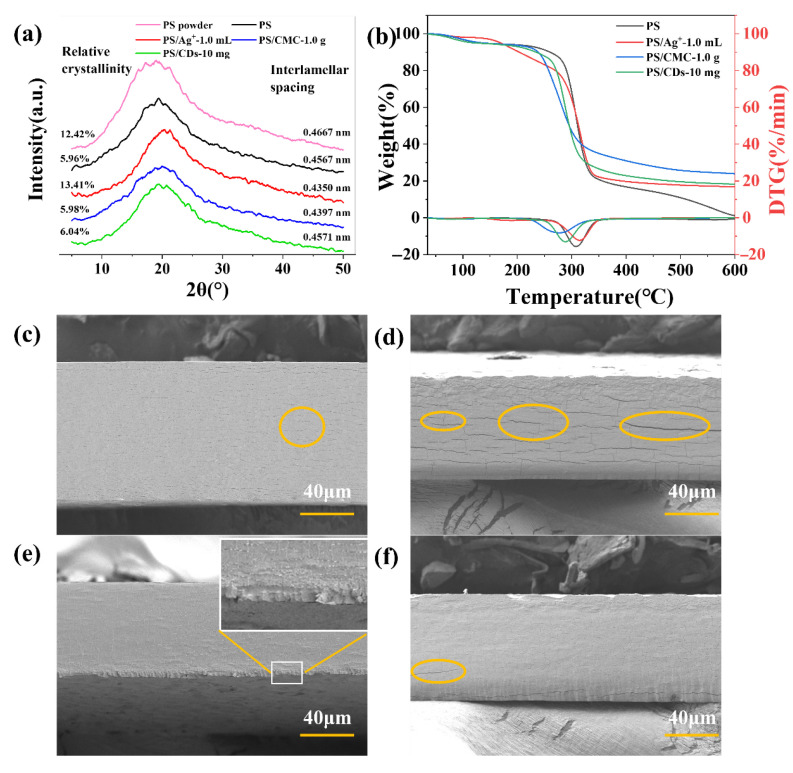
Property characterization of PS composite films. (**a**) Lattice parameters (XRD); (**b**) TG curve; (**c**) SEM of pristine PS films (**d**) SEM of PS/Ag^+^ composite film; (**e**) SEM of PS/CMC composite film; (**f**) SEM of PS/CDs composite film.

XRD analysis reveals distinct effects of different modifiers on the crystal structure of phosphate starch (PS) films (Figure 4a). The crystallinity of pristine PS powder measures 12.42%, which decreases to 5.96% after film formation, indicating a partial disordering of the molecular arrangement during processing. The crystallinity of the PS/Ag^+^ composite film increased to 13.41%, with its lattice spacing reduced to 0.4350 nm (compared to 0.4667 nm for the pure PS film). This enhancement is attributed to coordination interactions between Ag^+^ ions and phosphate groups, facilitating a more ordered molecular packing. The crystallinity values of PS/CMC and PS/CDs composite films are 5.98% and 6.04%, respectively, remaining comparable to that of the pristine PS film. This suggests that carboxymethyl cellulose and carbon dots interact primarily through hydrogen bonding and other non-covalent interactions without substantially altering the crystalline domains of PS.

Thermogravimetric analysis reveals distinct thermal behaviors among the films (Figure 4b). The thermal degradation of the starch-based film occurs in two distinct stages. The first stage (approximately 50–200 °C) is primarily attributed to the evaporation and volatilization of moisture from the sample. Specifically, weight loss below 100 °C corresponds to the removal of free water, while loss between 100 and 250 °C results from the release of bound water adsorbed within the polyhydroxy structure of the starch. The second stage (approximately 250–400 °C) corresponds to the thermal decomposition and carbonization of the starch molecular chains, carboxymethyl cellulose chains, and carbon dots [29]. The maximum degradation temperature of the pristine PS film is 308 °C. Among the composite films, PS/Ag^+^ exhibits the highest thermal stability, with its maximum degradation temperature increasing to 315.6 °C and a char residue of 16.8%, which is consistent with the enhanced crystalline ordering observed across the composites, as summarized in Table 1. In contrast, the PS/CMC film shows a reduced degradation temperature of 278.1 °C yet yields the highest char residue (23.9%). This behavior likely results from the relatively low intrinsic thermal stability of carboxymethyl cellulose chains and their tendency to undergo condensation during thermal decomposition. The PS/CDs composite degrades at 288.94 °C with 18.3% char residue, suggesting that hydrogen bonding between carbon dots and PS molecular chains contributes to a moderate improvement in thermal stability while helping to maintain material flexibility [30].

Scanning electron microscopy (SEM) was employed to analyze the fracture morphology of the films. The PS/CMC film exhibited a significant improvement in tensile strength, attributable to the reinforcing effect of cellulose’s two-dimensional network structure, and characteristics of ductile fracture were observed in its lower layer. Compared to the pure PS film (Figure 4c,e), the PS/Ag^+^ composite exhibits a greater number of elongated microcracks at its interfaces. This observation is consistent with the crystallization data (Figure 4a), which indicates that silver ions serve as nucleating agents, thereby enhancing the overall crystallinity of the composite film. However, the localized rigid microcrystals formed by Ag^+^ introduce the stress concentration sites. Under tensile loading, cracks readily initiate and propagate from the defects, resulting in the observed microcracks (Figure 4d). The fracture surface of the PS/CDs composite resembles that of the unmodified PS film, indicating uniform dispersion of carbon dots within the starch matrix. The hydroxyl and carboxyl groups on the carbon dot surface form hydrogen bonds with polar groups on the phosphate starch chains (Figure 3a), contributing to microcrystal formation. In regions with well-dispersed carbon dots, strong interfacial bonding is achieved via hydrogen bonding. However, in regions of carbon dot agglomeration, the internal cohesion within agglomerates exceeds the interfacial bonding with the starch matrix, creating localized weak interfaces. Upon stretching, stress concentrates at these defects, leading to the formation of micro-voids (Figure 4f), though to a lesser extent than in the PS/Ag^+^ film.

### 3.5. Effect of Modifiers on the Impedance of PH Films

**Figure 5:**
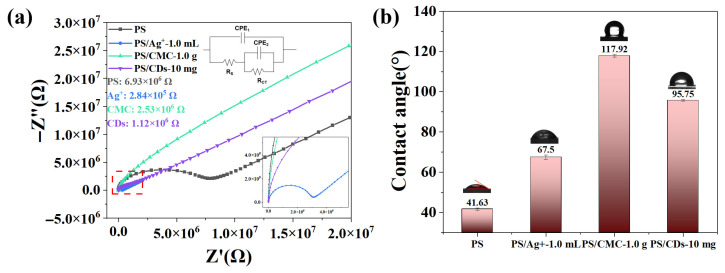
(**a**) EIS of the PS composite film; (**b**) Water contact angle of the PS composite film.

The charge transport properties of phosphate starch (PS) films modified with different additives were investigated by electrochemical impedance spectroscopy (EIS). Figure 5a shows the ZView software (3.0a) fitting results of the PS films, PS/Ag^+^ films, PS/CMC films and PS/CD films, with the corresponding equivalent circuit diagram plotted in the inset. The corresponding fitting results are summarized in Table 2. In general, a lower charge transfer resistance (R_CT_) represents a higher electron transport efficiency. The unmodified PS film exhibits a R_CT_ of 6.93 × 10^6^ Ω. With a water contact angle of 41.63° (Figure 5b), the film demonstrates hydrophilic character, which facilitates the penetration of external ions. Nevertheless, the overall impedance remains relatively high, which can be attributed to the limited ion dissociation capacity of PS and the absence of continuous electron conduction pathways.

The incorporation of different modifiers leads to notable variations in film impedance. The PS/Ag^+^ films have the lowest series resistance (R_S_) of 26.4 Ω, indicating higher electron extraction efficiency. The PS/Ag^+^ composite exhibits a substantial reduction in R_CT_ to 2.84 × 10^5^ Ω, which represents a decrease of approximately 96% compared to the unmodified film. This marked improvement is primarily attributed to the increased carrier concentration resulting from the dissociation of Ag^+^ ions. In contrast, the PS/CMC film shows a R_CT_ of 2.53 × 10^6^ Ω. The insulating nature of CMC restricts efficient charge transport, thereby maintaining a relatively high impedance. The PS/CDs composite demonstrates a R_CT_ of 1.12 × 10^6^ Ω, corresponding to an 84% reduction relative to the pristine PS film. This enhancement originates from the conjugated carbon core of the CDs, which provides effective electron transport pathways [31]. Simultaneously, the hydrogen-bonded crosslinked network formed between CDs and the starch matrix preserves moderate ionic conductivity. Consequently, a synergistic ion-electron transport mechanism is established, enabling significant impedance optimization without employing metal ions [32].

### 3.6. pH-Responsive Composite Films as a Platform for pH Sensing

**Figure 6:**
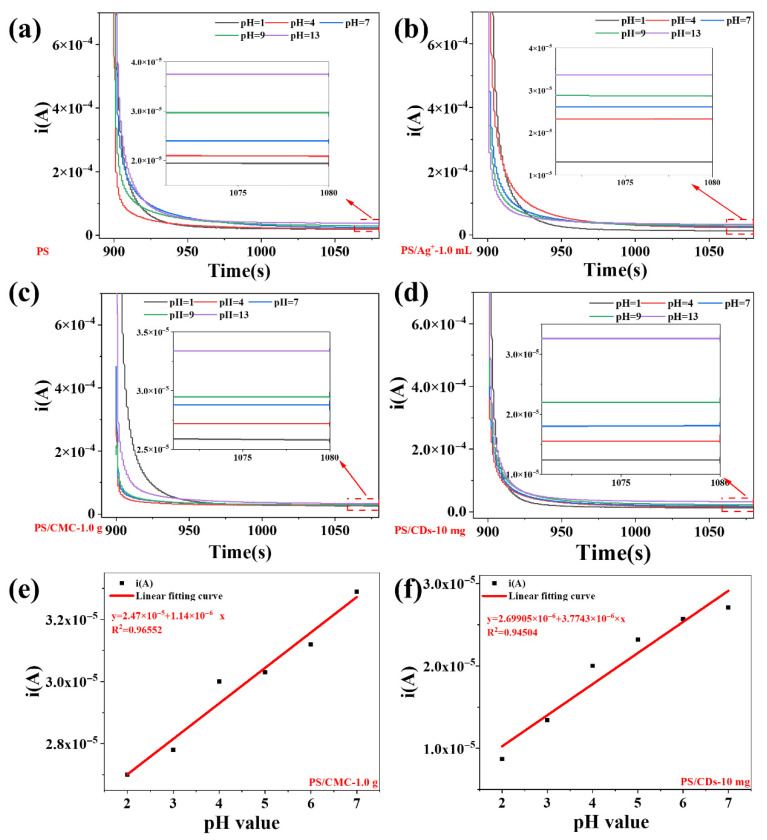
Chronoamperometric characterization of films in pH environment. (**a**) PS film; (**b**) PS/Ag^+^ composite film; (**c**) PS/CMC composite film; (**d**) PS/CDs composite film; (**e**) Current fitting curve for PS/CMC film; (**f**) Current fitting curve for PS/CDs film.

The chronoamperometric responses of the four composite films were systematically evaluated across varying pH environments (Figure 6). All films exhibited a distinct pH-dependent behavior: the steady-state current was moderate at neutral condition (pH = 7), decreased markedly in acidic environments, and increased significantly under alkaline conditions. Specifically, the steady-state current for the pristine PS film ranged from 1.93 × 10^−5^ A to 3.82 × 10^−5^ A, while the PS/CDs composite film showed a range of 1.22 × 10^−5^ A to 3.29 × 10^−5^ A.

Over the acidic pH range of 2–7, the PS/CMC and PS/CDs composite films display a strong linear correlation between current and pH value. The fitted linear equations yield determination coefficients (*R*^2^) of 0.9655 for PS/CMC and 0.9450 for PS/CDs, demonstrating that the current signal reliably tracks pH variations. This pH-responsive behavior is attributed to the protonation–deprotonation equilibrium of surface functional groups (e.g., carboxyl and hydroxyl) [33]. The abundant oxygen-containing moieties on the carbon dots contribute notably to the enhanced sensitivity [34]. Given that most liquid food products exhibit pH values in the acidic-to-neutral range, these composite films offer a promising platform for the development of electrochemical sensors aimed at food freshness monitoring and acidity detection [31].

### 3.7. Application of PS/CDs Composite Films in Juice Spoilage Detection

**Figure 7:**
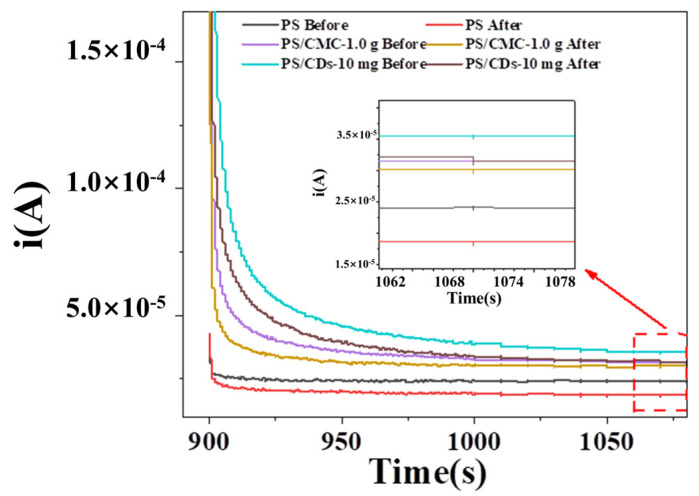
Chronoamperometric curves of composite films from pH environment to juice detection.

To evaluate the potential of composite films for quality monitoring of liquid beverages, fresh (pH = 3.38) and spoiled (pH = 2.68) orange juice were selected as model systems. The electrochemical response of three film types was assessed by chronoamperometry (Figure 7). The steady-state current of the pristine PS film measured 2.39 × 10^−5^ A for fresh juice and 1.91 × 10^−5^ A for spoiled juice, corresponding to a current change (ΔI) of 0.48 × 10^−5^ A. In contrast, the PS/CMC composite film showed currents of 3.21 × 10^−5^ A (fresh) and 3.20 × 10^−5^ A (spoiled), with only a minimal ΔI, indicating negligible pH responsiveness in this acidic range. Notably, the PS/CDs composite film demonstrated the most distinct current differentiation, recording 3.56 × 10^−5^ A for fresh and 2.94 × 10^−5^ A for spoiled juice, yielding a ΔI of 0.62 × 10^−5^ A. This enhanced signal disparity underscores the superior discrimination capability of the PS/CDs film toward juice spoilage.

The observed differences in current response can be attributed to the distinct charge transport mechanisms of the materials. The pristine PS film primarily relies on H^+^ dissociation from phosphate groups for ionic conduction, whereas its highly tortuous ion migration pathways result in significantly elevated impedance. In contrast, the conjugated carbon cores in CDs establish continuous electron transport pathways, which operate synergistically with the ionic conduction network of PS to form a hybrid ion–electron conduction mechanism. This dual conduction system substantially enhances overall charge transfer efficiency, thereby enabling the PS/CDs composite film to achieve superior sensitivity in detecting juice spoilage.

### 3.8. Fluorescence Properties and pH Response Mechanism of PS/CDs Composite Film

**Figure 8:**
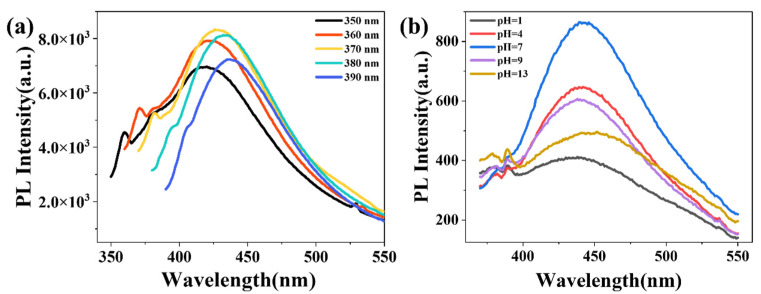
Fluorescence properties of the PS/CDs composite film. (**a**) Excitation spectrum; (**b**) pH-responsive fluorescence spectra.

The fluorescence properties of the PS/CDs composite films and their pH-responsive behavior were investigated by analyzing their excitation spectra and fluorescence emission under different pH conditions. The excitation spectra (Figure 8a) showed a gradual red shift in the emission maximum as the excitation wavelength increased from 350 to 390 nm, with the fluorescence intensity first increasing and then decreasing. The most intense blue emission was observed at 427 nm under an excitation wavelength of 370 nm.pH-dependent fluorescence measurements (Figure 8b) revealed that the fluorescence intensity was maximal at neutral pH (7) but was significantly reduced under both acidic (pH 1, 4) and alkaline (pH 9, 13) conditions. This response was most pronounced in the strongly acidic range (pH 1–4). This behavior is attributed to the protonation–deprotonation equilibria of surface functional groups (e.g., carboxyl and hydroxyl) on the carbon dots. At neutral pH, these groups exist in a balanced charge state that facilitates efficient radiative recombination (fluorescence emission). In contrast, under acidic or alkaline conditions, protonation or deprotonation disrupts this balance, inducing surface charge redistribution which promotes non-radiative decay pathways and, consequently, leads to the observed fluorescence quenching.

In the conduction mechanism, the phosphorus component serves as the foundational matrix for ion transport, while the conjugated carbon cores of the CDs provide a percolating network for electrons. Together, they create an effective dual-pathway network that effectively lowers the overall film impedance. This CD-based strategy is particularly advantageous, as in contrast to other modifiers, it achieves a multifunctional combination of low impedance, high toughness, and pH-responsive fluorescence without incorporating metal ions, thereby demonstrating a novel and sustainable approach for developing green starch-based sensing materials.

### 3.9. Long-Term Fluorescence Experiment of the PS/CDs Composite Film

**Figure 9:**
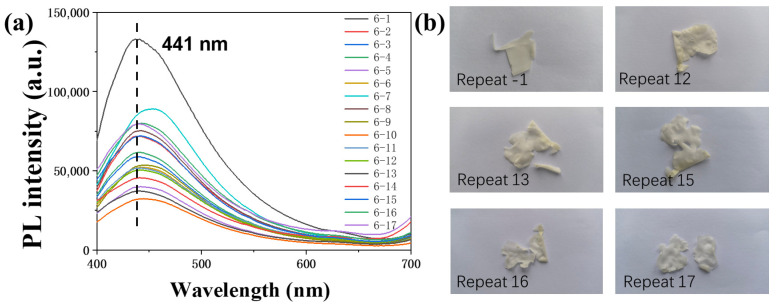
(**a**) Fluorescence spectra of the PS/CDs film upon repeated testing; (**b**) Optical images of the CA/CDs film after various testing cycles.

**Figure 10:**
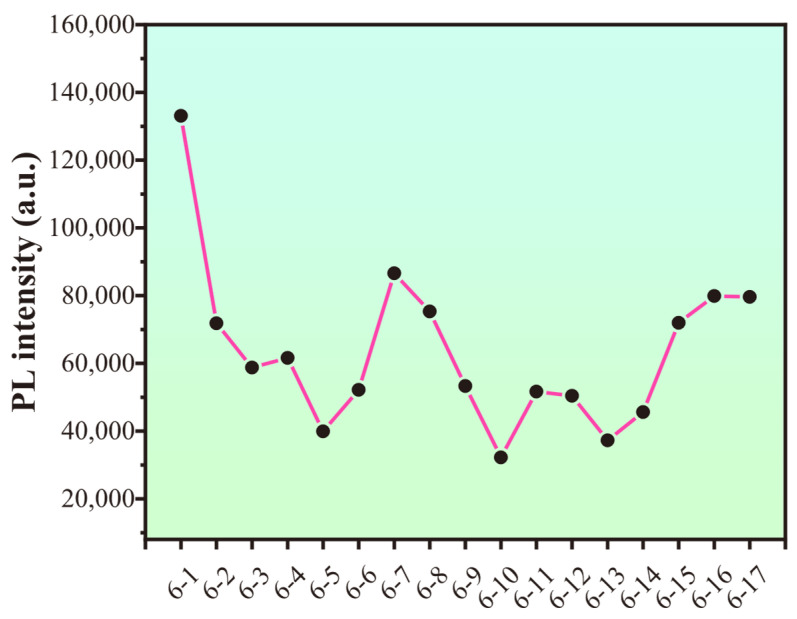
Variation in CA/CDs composite film fluorescence intensity over cycles.

The PS/CDs film suffered structural failure at the 12th cycle. Testing was continued until the 17th cycle (Figure 9), at which point the material had softened excessively and the experiment was terminated. The fluorescence intensity showed a correlation with the number of cycles, exhibiting a general declining trend as testing progressed (Figure 10).

**Figure 11:**
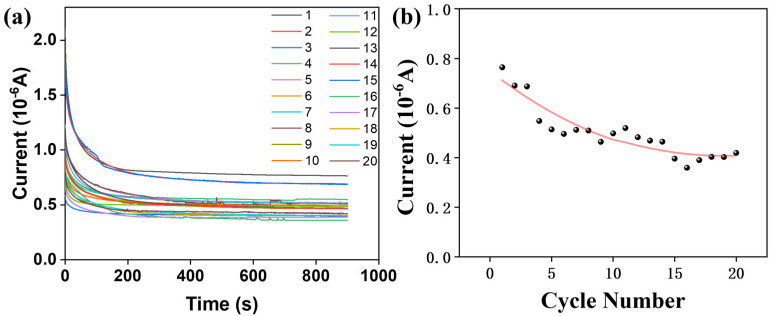
(**a**) PS/CDs composite thin film electrical signal curve; (**b**) Trend in PS/CDs composite film electrical signal over test cycles.

To evaluate the long-term performance, the composite film-based devices were subjected to 20 consecutive cycling tests using a pH = 3 solution to monitor the trend in electrical signal response (Figure 11a). For the PS/CDs composite film, the electrical signal decreased progressively with an increasing number of cycles (Figure 11b), indicating a decline in device sensitivity upon repeated use.

## 4. Conclusions

This chapter systematically examines the effects of three modifiers (Ag^+^, CMC, and CDs) on the properties of phosphate starch films. The findings reveal distinct interfacial engineering mechanisms: Ag^+^ ions strengthen the film via coordination cross-linking, albeit at the expense of toughness. CMC forms an extensive hydrogen-bonding network, significantly enhancing strength but inducing brittle fracture. In contrast, CDs facilitate a synergistic enhancement of both strength and toughness through nanoscale, flexible cross-linking.

The incorporation of carbon dots (CDs) enhances the strength of the composite film, while maintaining toughness comparable to that of the unmodified matrix. This strengthening effect is attributed to hydrogen bonding between the CDs and the phosphate starch, which facilitates an increase in film crystallinity.

Functionally, carbon dots (CDs) imparted a range of enhanced properties to the composite film. The establishment of a dual conduction pathway for ions and electrons reduced the film’s electrical impedance from 2.03 × 10^6^ Ω to 1.12 × 10^6^ Ω, corresponding to a decrease of approximately 45%. Concurrently, the thermal stability was significantly improved, as evidenced by the char residue increasing from 1.1% (pristine PS) to 18.3%. Furthermore, the PS/CDs film exhibited a linear current response over the pH range of 2–7 (*R*^2^ = 0.9450). A measurable current change of 0.62 × 10^−5^ A during juice spoilage underscores its potential for practical sensing applications, although limitations such as a relatively narrow scope of application and unconfirmed long-term stability remain to be addressed in future studies.

This study investigates the synergistic enhancement mechanism between phosphate groups and carbon dots from multiple perspectives, thereby providing a theoretical basis for the structural design and performance optimization of starch-based smart packaging materials.

## Figures and Tables

**Table 1:** Maximum weight loss rate temperature (Tp) and residual carbon rate (RC) of the composite films.

Sample	Tp (°C)	RC (%)	T_1_%	T_5_%	T_10_%
PS	308.0	1.1	68.1	156.1	255.3
PS/Ag^+^-1.0 mL	315.6	16.8	70.3	170.6	203.2
PS/CMC-1.0 g	278.1	23.9	71.4	138.9	238.0
PS/CDs-10 mg	288.9	18.3	57.9	122.3	234.5

**Table 2:** ZView2 fitting parameters obtained from the electrochemical impedance spectra data of the PS films, PS/Ag^+^ films, PS/CMC films and PS/CD films.

Samples	R_S_ (Ω)	R_CT_ (Ω)	CPE_1_	CPE_2_
PS	42.7	6.93 × 10^6^	9.16 × 10^−9^	9.75 × 10^−9^
PS/Ag+	26.4	2.84 × 10^5^	8.94 × 10^−9^	9.63 × 10^−9^
PS/CMC	32.5	2.53 × 10^6^	9.02 × 10^−9^	9.56 × 10^−9^
PS/CD	28.3	1.12 × 10^6^	9.07 × 10^−9^	9.69 × 10^−9^

## Data Availability

The data presented in this study are available on request from the corresponding author due to legal and intellectual property considerations related to an ongoing patent application.

## References

[1] Musahgi K.U., Oliveira G., Ahmad M., Mustafa A., Herculano R.D., Farias F. (2024). Halochromic properties of carotenoid films for smart food packaging. Food Packag. Shelf Life.

[2] Bomic S., Agyei D., Ali A. (2024). Smart chitosan films as intelligent food packaging: Approaches for monitoring food freshness and biomarkers. Food Packag. Shelf Life.

[3] Liu Z., Li N., Niu L., Xu L., Feng J., Liu Z. (2025). Eco-friendly smart packaging films with high thermal stability, antibacterial activity, and food freshness monitoring. Food Bioprocess Technol..

[4] Syed N.S., Howell N.K., Sarbon N.M. (2023). A Review on Potential Use of Gelatin-based Film as Active and Smart Biodegradable Films for Food Packaging Application. Int. J. Food Sci. Technol..

[5] Zolek-Tryznowska Z., Kaluza A. (2021). The influence of starch origin on the properties of starch films: Packaging performance. Materials.

[6] Zhang L., Zhao J., Li F., Xu J., Yang B., Li Q. (2024). Influence of amylose and amylopectin fine structure on the thermo-mechanical and hydrophobic properties of starch films. Int. J. Biol. Macromol..

[7] Liu W., Chen L., McClements D.J., Peng X., Xu Z., Jin Z. (2024). Development of starch films for value-added utilization of starch in food and biomedical fields. Food Biosci..

[8] Yang F., Wei Y., Xiao H., Zhang Q., Li J., Lin Q., Zhu D., Huang Z., Liu G. (2023). Acetylated rice starch nanocrystals improve the physical, mechanical and structural properties of native starch-based films. Int. J. Biol. Macromol..

[9] Kalu A.O., Omonije O.O., Egwim E.C., Jigam A.A., Muhammad H.L. (2025). Crosslinking modification for starch and starch-based films (A review). Starch-Stärke.

[10] Cahyana Y., Verrell C., Kriswanda D., Aulia G.A., Yusra N.A., Marta H., Sukri N., Esirgapovich J.S., Abduvakhitovna S.S. (2023). Properties comparison of oxidized and heat moisture treated (HMT) starch-based biodegradable films. Polymers.

[11] Huang X., Chen L., Liu Y. (2024). Effects of ultrasonic and ozone modification on the morphology, mechanical, thermal and barrier properties of corn starch films. Food Hydrocoll..

[12] Dhanyasree P., Neenu K.V., David D.A., Begum P.M.S., Yoosaf K. (2024). An eco-friendly polymer-silver nanocomposite film for simultaneous sensing and removal of mercury from water. Microchem. J..

[13] Vieira I.R.S., Carvalho A.P.A., Conte-Júnior C.A. (2022). Recent advances in biobased and biodegradable polymer nanocomposites, nanoparticles, and natural antioxidants for antibacterial and antioxidant food packaging applications. Compr. Rev. Food Sci. Food Saf..

[14] Ramakrishnan R., Kulandhaivelu S.V., Roy S., Viswanathan V.P. (2023). Characterisation of ternary blend film of alginate/carboxymethyl cellulose/starch for packaging applications. Ind. Crops Prod..

[15] Lin L., Peng S., Chen X., Li C., Cui H. (2023). Silica nanoparticles loaded with caffeic acid to optimize the performance of cassava starch/sodium carboxymethyl cellulose film for meat packaging. Int. J. Biol. Macromol..

[16] Amaregouda Y., Kamanna K. (2024). Carboxymethyl cellulose/starch-based films incorporating chitosan nanoparticles for multifunctional food packaging. Cellulose.

[17] Qin S., Sun H., Wan X., Wu Y., Lin X., Kan H., Hou D., Zheng Z., He X., Liu C. (2023). Carboxymethylcellulose reinforced starch films and rapid detection of spoiled beverages. Front. Bioeng. Biotechnol..

[18] Yorozuya H., Ashrafi N.E., Sato K. (2025). Synthesis and fluorescence mechanism of nitrogen-doped carbon dots utilizing biopolymer and urea. Molecules.

[19] Zhang L.Q., Sun R., Liang Y.H. (2025). Carbon quantum dot dual-regulation for constructing high-performance NFPP cathode: Synergistic breakthrough in electron conductivity and ion transport. Energy Storage Mater..

[20] Afonso A.C.P., Correia A.S., Duarte D., Brandão A.T.S.C., Martínez de Yuso M.d.V., Jiménez-Jiménez J., Vale N., Pereira C.M., Algarra M., Pinto da Silva L. (2021). An active surface preservation strategy for the rational development of carbon dots as pH-responsive fluorescent nanosensors. Chemosensors.

[21] Kumari M., Chaudhary G.R., Chaudhary S., Huang M., Guo Z. (2024). Transformation of waste rice straw to carbon quantum dots and their potential chemical sensing application: Green and sustainable approach to overcome stubble burning issues. Biorefinery.

[22] Zasada L., Świątek M., Cieśliska M., Olewnik-Kruszkowska E., Kaczmarek-Szczepańska B. (2025). Enhancement of chitosan-based films for blueberries packaging- by modification with ellagic acid and cinnamic acid. Polym. Degrad. Stab..

[23] Brzęczyska M.Ś., Hindе A.H., Kaczmarek-Szczepańska B., Jankiewicz U., Urbańiak J., Boryński S., Zasada L., Cieśliska M., Dębińska K., Palubicka K. (2024). Biodegradability Study of Modified Chitosan Films with Cinnamic Acid and Ellagic Acid in Soil. Polymers.

[24] Stelescu M.D., Oprea O.-C., Sonmez M., Ficai A., Motelica L., Ficai D., Georgescu M., Gurau D.F. (2024). Structural and Thermal Characterization of Some Thermoplastic Starch Mixtures. Polysaccharides.

[25] Erden R.F., Avcilar F.K., Beyhan S. (2025). Starch-based biopolymer films with nitrogen-doped carbon quantum dots for enhanced barrier functions via surface microarchitectures. Int. J. Biol. Macromol..

[26] Xu H., Cheng H., McClements D.J., Chen L., Long J., Jin Z. (2022). Enhancing the physicochemical properties and functional performance of starch-based films using inorganic carbon materials: A review. Carbohydr. Polym..

[27] Zhao L.L., Jiang H.N., Han Z.X., Gu W.Q., Meng X.R. (2025). Starch coating with carbon dots: A promising coating to enhance the freeze-thaw stability of meatballs. Food Chem. X.

[28] Koshy R.R., Koshy J.T., Mary S.K., Sadanandan S., Jisha S., Pothan L.A. (2021). Preparation of pH sensitive film based on starch/carbon nano dots incorporating anthocyanin for monitoring spoilage of pork. Food Control.

[29] Liu Z.B., Cui M., Weng R., Li H.B., Hati S., Hu L.B., Mo H.Z. (2024). Incorporation of carbon dots into polyvinyl alcohol/corn starch based film and its application on shiitake mushroom preservation. Int. J. Biol. Macromol..

[30] Wu Y.Q., Zhang J.J., Hu X.T., Huang X.W., Zhang X.A., Zou X.B., Shi J.Y. (2024). Preparation of edible antibacterial films based on corn starch /carbon nanodots for bioactive food packaging. Food Chem..

[31] Javanbakht S., Namazi H. (2017). Solid state photoluminescence thermoplastic starch film containing graphene quantum dots. Carbohydr. Polym..

[32] Li R.H., Yang Y.J., Li C.C., Liao J.Z., Zhu S.Q., Liu R., Zhu H.J. (2025). Synthesis of starch-based F/N-doped carbon dots for enhanced detection of latent fingerprint tertiary structures. New J. Chem..

[33] Preethi M., Murugan R., Viswanathan C., Ponpandian N. (2022). Potato starch derived N-doped carbon quantum dots as a fluorescent sensing tool for ascorbic acid. J. Photochem. Photobiol. A Chem..

[34] Yadav N., Mudgal D., Mishra A., Shukla S., Malik T., Mishra V. (2024). Harnessing fluorescent carbon quantum dots from natural resource for advancing sweat latent fingerprint recognition with machine learning algorithms for enhanced human identification. PLoS ONE.

